# Rationale and design of the PaTIO study: PhysiotherApeutic Treat-to-target Intervention after Orthopaedic surgery

**DOI:** 10.1186/s12891-020-03511-y

**Published:** 2020-08-14

**Authors:** Lichelle Groot, Maaike G.J. Gademan, Wilfred F. Peter, Wilbert B. van den Hout, Hennie Verburg, Thea P.M. Vliet Vlieland, Max Reijman, on behalf of the PaTIO study group, D. C. Baas, R. Bazuin, K. Boerma-Argelo, B. Boonen, P. K. Bos, E. A. Breedveld, M. de Bruijn, B. Dijkstra, J. Elings, A. de Gast, T. Gosens, D. J. Hofstee, R. P. A. Janssen, L. Jutten-Brouwer, P. M. van Kampen, H. Kaptijn, S. Koëter, C. A. L. C. Kremers-van de Hei, W. Y. Liu, A. F. Lenssen, M. F. Nieboer, L. Nieuwenhuys-Kroon, P. A. Nolte, J. C. A. Noorduyn, E. Oosting, J. H. Pasma, R. W. Poolman, M. Schager, M. G. M. Schotanus, R. J. A. Sonnega, M. Stevens, S. H. M. Verdegaal, W. P. Zijlstra

**Affiliations:** 1 Department of Orthopedic Surgery, Erasmus MC, University Medical Center Rotterdam, P.O. Box 2040, 3000 CA Rotterdam, Netherlands; 2 Department of Orthopedic Surgery, LUMC, Leiden University Medical Center, P.O. Box 9600, 2300 RC Leiden, The Netherlands; 3 Department of Clinical Epidemiology, LUMC, Leiden University Medical Center, P.O. Box 9600, 2300 RC Leiden, The Netherlands; 4 Department of Medical Decision Making & Quality of Care, LUMC, Leiden University Medical Center, P.O. Box 9600, 2300 RC Leiden, The Netherlands; 5 Department of Orthopedic Surgery, Reinier de Graaf Hospital, Reinier de Graafweg 5, 2625 AD Delft, The Netherlands

**Keywords:** Physiotherapy modalities, Arthroplasty, replacement, knee, Arthroplasty, replacement, hip, Cost-benefit analysis, Randomized controlled trial

## Abstract

**Background:**

Physiotherapy is a proven effective treatment strategy after total knee and hip arthroplasty (TKA/THA), however there is considerable practice variation regarding its timing, content and duration. This study aims to compare the (cost-) effectiveness of a standardized, treat-to-target postoperative physiotherapy strategy with usual postoperative care.

**Methods:**

Using a cluster randomized study design, consecutive patients scheduled for a primary TKA/THA in 18 hospitals in the Netherlands will be assigned to the treat-to-target physio therapy strategy or usual postoperative care. With the treat-to-target strategy a standardized, individually tailored, exercise program is aimed at the attainment of specific functional milestones. Assessments are done at baseline, 6 weeks and 3, 6, 9 and 12 months follow up. The primary outcome will be the Knee injury / Hip disability and Osteoarthritis Outcome Score - Physical Function Short Form (KOOS-PS / HOOS-PS) at 3 months follow up. Secondary outcomes are the numeric rating scale for pain, the Oxford Knee and Hip Scores, performance-based test and the EuroQol 5D-5L for quality of life. Healthcare use, productivity and satisfaction with postoperative care are measured by means of questionnaires. In total, 624 patients will be needed of which 312 TKA and 312 THA patients.

**Discussion:**

The study will provide evidence concerning the (cost-) effectiveness of the treat-to-target postoperative physiotherapy treatment compared to usual postoperative care. The results of this study will address an important evidence gap and will have a significant impact in daily practice of the physio therapist.

**Trial registration:**

Registered in the Dutch Trial Registry on April 15, 2018. Registration number: NTR7129.

## Background

In patients undergoing total knee or hip arthroplasty (TKA/THA) for osteoarthritis (OA) postoperative physiotherapy (PPT) is a recommended treatment [1–4]. Several studies have demonstrated its effectiveness in improving function, range of motion and quality of life [5–8]. Moreover, PPT also seems to play an important role in achieving functional independence such as return to work [9, 10].

Despite the current evidence, there is no consensus on the optimal composition of the treatment, timing (when to start and stop), and dosage (frequency). This has led to considerable practice variation, as demonstrated by a number of studies [11–13]. For instance, a Dutch multicenter study on 522 TKA and THA patients found variation in the use of active and passive treatment modalities, and large individual differences in duration of treatment [14]. Potential sources of variation are constituted by different postoperative treatment protocols used by orthopedic surgeons and hospital physio therapists (PTs) [15], as well as variation among primary care PTs delivering the intervention. About 1.66 and 1.26 per 1000 people receive TKA or THA respectively in western countries each year [16]. From the health care and societal perspective, it is highly relevant that the most optimal PPT treatment strategy is used. Therefore, more knowledge on optimized PPT strategies is necessary [16, 17].

It is conceivable that PPT may be optimized by using a standardized treatment, that is tailored to the patient’s individual situation. Previous research has shown that targeting treatment using specific milestones, also called treat-to-target strategy, was found to assist physicians in disease management, by simplifying and facilitating disease management decisions [18]. It is proven effective in improving clinical outcomes in various chronic conditions such as, cardiovascular disease and rheumatoid arthritis [12, 19, 20]. However, milestones are currently not routinely used in PPT after TKA or THA to direct treatment. Another way via which care can be optimized is by use of a transmural care pathway, which includes a short stay (1–2 days) and acute (secondary care) and post-acute care (primary care). In TKA and THA these transmural pathways are known to result in less postoperative complications, shorter length of hospital stay and lower costs during hospital stay when compared to standard care (3–4 nights inpatient) [21–23]. Hence, a transmural pathway for PPT after TKA and THA linking all organizational aspects related to the continuum of TKA/THA care might also improve PPT. As such, we propose an individualized PPT intervention that is relatively standardized, yet the precise content, frequency and duration of treatment are determined by the acquisition of individualized, functional milestones. This research topic was prioritized on the research agenda of the Dutch Orthopaedic Association (NOV). Within this project, the NOV and the Royal Dutch Society for Physio therapy (KNGF) collaborate.

## Trial objectives

The aim of the present study is to evaluate the (cost-) effectiveness of a standardized, treat-to-target PPT strategy in TKA and THA patients compared to usual PPT. We hypothesize that with the standardized treat-to-target strategy better functional outcome can be achieved compared to usual care with lower costs (superiority study). The results of this study will be used to develop and implement a nationwide treatment strategy for PT after TKA/THA, and will be integrated in national guidelines of PTs and orthopedic surgeons.

Primary objective

1. To assess whether the functional outcome of a standardized, personalized treat-to-target PT strategy after TKA and THA is superior to usual care PPT after 3 months follow-up.
2. To assess whether a standardized, personalized treat-to-target PPT strategy is more cost-effective compared to usual care PPT after 12 months follow-up.

Secondary objective

1. To assess whether the functional outcome of a standardized, personalized treat-to-target PPT strategy after TKA and THA is superior to usual care PPT during the follow-up of 12 months.
2. To assess the difference in function, performance-based tests, pain and quality of life, as well as anchor questions, and satisfaction question; between both groups over time (6 weeks, 3,6,9 and 12 months) during the first year.

## Methods/design

### Study design and setting

We will use a multicenter, cluster randomized design. The 18 active including centers are; Alrijne hospital (Leiderdorp), Bergman Clinics (Rijswijk), Canisius Wilhelmina hospital (Nijmegen), Diakonessenhuis (Utrecht), Elisabeth-Tweesteden hospital (Tilburg), Erasmus MC University Medical Center (Rotterdam), HAGA hospital (Den Haag), hospital Gelderse Vallei (Ede), Leiden University MC (Leiden), Máxima MC (Eindhoven), MC Leeuwarden, Northwest Clinics (Alkmaar) OLVG (Amsterdam), Spaarne hospital (Hoofddorp), Tergooi hospital (Hilversum), University MC Groningen, University MC Maastricht and Zuyderland MC (Sittard). Lange Land hospital (Zoetermeer) and Reinier de Graaf Gasthuis (Delft) are participating in the study however they are not including patients. Reinier de Graaf is involved in developing the PPT protocol and a part of the patients of HAGA hospital will have surgery in Lange Land hospital.

In the intervention group, PT is delivered by selected primary care practices by specially trained PTs. The training is a physical training and will be given by the same project members. In the control group the choice to use PT and the primary care practice is left to the discretion of the orthopaedic surgeon and the patient. In the Netherlands, after TKA or THA, a referral from the orthopaedic surgeon is needed for the healthcare insurance. This referral is standardly given by discharge from the hospital.

The planned duration of the study is three years.

The study protocol was reviewed and approved by the Erasmus MC Medical Ethics Committee (registration code NL61763.078.17) and was registered in the Dutch Trial Registry (registration number NTR7129). In all participating hospitals, the local responsible authorities approved the conduct of the study.

### Study population

#### Inclusion and exclusion criteria

##### Inclusion

Patients eligible for this trial are; a. patients with clinical and radiological knee or hip OA who are scheduled for a primary TKA or THA, b. willing to comply with the study protocol and c. providing written informed consent.

##### Exclusion

Patients with a TKA or THA for a diagnosis other than OA; b. uncontrolled cardiovascular disease or hypertension; c. history of neuromuscular disorder that affects lower extremity function, d. terminal illness; e. planned replacement of another joint during study follow-up; f. not able to attend follow-up measurements; g. not able to attend the PPT in primary setting; h. serious psychiatric disorders; i. insufficient command of the Dutch language, spoken and/or written.

#### Recruitment

Patients who are scheduled for a primary TKA or THA in one of the participating hospitals and meet the eligibility criteria will be informed about the study by means of oral and written information. After written consent, baseline measurements are carried out. Recruitment is done by an orthopedic surgeon or researcher on his/her behalf.

### Randomization

To avoid contamination between treat-to-target intervention and usual care, randomization will take place on hospital level.

A randomization list was created by an independent statistician using Sealed Envelope (London, UK). Random permuted blocks sizes of 4, 6 and 8 were used, and randomization was stratified by type of hospital (academic or general). Upon request of the trial management, the independent statistician randomized a site using the randomization list and communicated the site allocated treatment strategy by e-mail. The trial management remained blind to the randomization throughout the study.

#### Blinding

The study is an open-label trial.

### Intervention

#### Development treat-to-target intervention

**Fig. 1:**
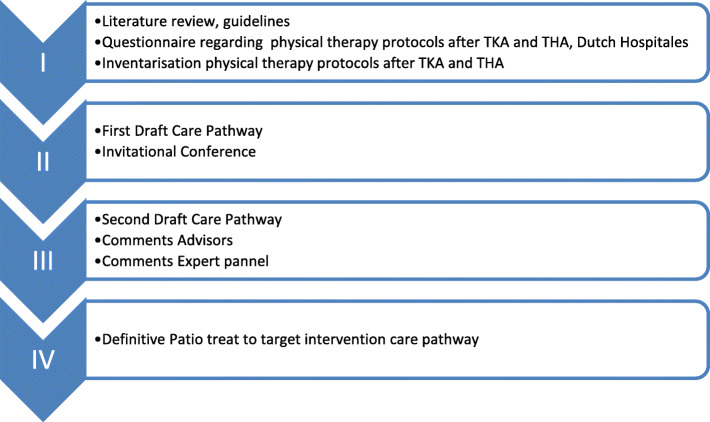
Development PATIO treat to target intervention care pathway

The treat-to-target PPT is presented in the form of a transmural care pathway and was developed in four different phases knowingly, 1) an inventory phase, 2) development of the first draft, 3) development second draft, 4) finalizing treat-to-target PPT (Fig. 1). In the first phase, a literature study on the efficacy of PPT was done, in which guidelines regarding THA and TKA were taken into account. Also, an assessment of the existing perioperative treatment protocols in the Netherlands was conducted. Based on a selection of best practice (integrality and evidence based), one protocol was selected as the fundament for our treat-to-target intervention. In the second phase this protocol was adapted and supplemented using literature and guidelines. Hereafter the first concept of the protocol was discussed in an expert meeting of one primary and one secondary PT, a patient and orthopedic surgeons. In the third phase, the concept protocol was adjusted and details regarding the implementation in daily practice were added by 2 PTs who have extensive experience in the field of developing treatment protocols. The second concept of the protocol was again presented to the PTs, the patient and orthopedic surgeons who attended the first expert meeting for feedback. In the fourth and last phase, the protocol was adjusted based on the feedback of the expert panel resulting in final our treat-to-target intervention. This intervention consists of a preoperative assessment by the primary PT, secondary PPT and a comprehensive and personal transferal from the hospital PT including a Modified Iowa Levels of Assistance Scale (MILAS) score as a starting point of the secondary PPT.

#### Intervention arm: treat-to-target intervention

First the necessity of PPT will be screened by a standardized assessment of the patient’s health status, personal-, external factors and achievement of functional milestones. This assessment will be performed by the primary care PT in the first appointment after surgery (within 3 days after discharge from the hospital). Those needing PPT in primary care receive a standardized, personalized, time contingent program, focused on evidence-based components such as muscle strengthening and functional exercises. Exercises, selected by the PT, are available via an app or printed by the PT via Physitrack, an online available exercise program. The progress of functioning of the patient will be regularly evaluated regarding achievement of functional milestones. After reaching the milestones, PPT is ended and patients will receive a tailored advice with home-based exercises in combination with referral to exercise activities in the community.

#### Control arm: usual care

The use of PT and the choice of the physical therapist, is left to the discretion of the treating physician and the patient.

### Assessments

Patients will be asked to fill in questionnaires at baseline, and at 6 weeks, 3- (primary end-point), 6-, 9 and 12-months follow-up. Performance-based tests will be performed at baseline, 6- and 12 months (see Table 1 for an overview). In each participating hospitals an assessor will be trained by the coordinating researcher to perform these Performance-based tests.

**Table 1:** Overview of measurement PATIO study

Timepoint	Allocation	Enrolment	Post-allocation	Close-out
	Randomization	Baseline, at inclusion	6 weeks,3, 6, 9 –months follow-up	12-months follow-up
Enrolment				
Eligibility screen		X		
Informed consent		X		
Allocation	X			
Interventions				
*Intervention group: Treat to target*			X	X
*Control group:* *Usual care*			X	X
ASSESSMENTS:				
*Demographic;* age, gender, side affected, BMI, education level, comorbidity		X		
*HOOS-PS / KOOS - PS*		X	X	X
*OHS / OKS*		X	X	X
*NRS*		X	X	X
*AAQ*		X	X (only at 3 months)	X
*EQ 5-D5L*		X	X	X
*Satisfaction question*			X	X
Cost-effectiveness			X	X
*Performance-based tests*		X	X (only at 3 months)	X
*(Serious) Adverse events*			X	X

#### Patient characteristics

At baseline the following patient characteristics will be obtained: age, sex, side affected, height, weight, education level, ethnical background, living situation (living alone, living with partner, living with partner and child (ren) and living with children), duration of complaints, previous surgery and existing comorbidities.

### Primary outcomes

#### Physical functioning

Physical functioning, assessed by the 5-item short version of the validated Dutch translation of the Hip disability and Osteoarthritis Outcome Score (HOOS) [24, 25] or the 7-item short version of the Dutch translation of the knee injury and Osteoarthritis Outcome Score (KOOS) questionnaire [26, 27]. Function scores will be determined on a scale from 0 (severe impairments in function) to 100 (no impairments in function).

### Direct and indirect costs

Medical consumption (visits to health care providers and usage of prescribed or over the counter medication), absence from work or decreased productivity at work, and patient costs to be able to do cost-effectiveness analysis will be assessed with a questionnaire at 3,6,9 and 12 months postoperative. Our questionnaire is based on the questionnaire for Costs associated with Psychiatric Illness (TiC-P) from the institute of medical technology assessment [28]. We have left out the irrelevant questions about psychiatric consultations. We added questions about PT consultation (frequency) and specified medication in use of pain medication.

### Secondary outcomes

#### Pain and functioning

##### Performance-based test

Performance-based test as recommended by Osteoarthritis Research Society International (OARSI) [29] namely, 30 s “chair-stand test”, “40m walk test” and “stair negotiation” test, will be evaluated by the assessor. Assessors in all participating hospitals are trained in using a Dutch translated OARSI protocol. These tests will be performed at baseline, 3 months and 12 months.

### Self-reported pain and function

#### Numeric Rating Scale

Hip and knee pain severity in rest and during activities in the past week will be assessed by the Numeric Rating Scale (NRS) [30]. Scores range between 0 (no pain) and 10 (worst pain imaginable).

#### Oxford Hip score / Oxford Knee Score

Pain intensity and functional limitations in daily activities will be assessed by the Oxford Hip score (OHS) [31] and the Oxford Knee Score (OKS) [32]. Both consist of 12 questions; each question will be scored from 0 to 4 with 4 being the best outcome. Overall scores running from 0 to 48 with 48 being the best functional outcome.

#### The Animated Activity Questionnaire (AAQ)

Basic daily activity limitations will be assessed with the Animated Activity Questionnaire (AAQ) [33] in which patients see video animations of 17 different basic daily life activities. These activities closely mimic real-life situations. For each activity, three to five different levels of difficulty in performing the activity are shown as video animations at the same time. The patient is asked to choose the video that best matches his or her own performance. Function scores will be determined on a scale from 0 (severe impairments in function) to 100 (no impairments in function). This video animated questionnaire will be assessed during the physical assessment appointment at baseline, 3 months and 12 months.

### Quality of life

#### EuroQol-5D-5L

Health-related quality of life will be assessed by the EuroQol-5D-5L (EQ-5D-5L) [34]. This questionnaire consists of five domains (mobility, self-care, daily activities, pain/discomfort, and anxiety/depression) in which the patient can self-rate their severity of problems on a 5-point scale ranging from no problems to extreme problems or unable to perform the task.

### Postoperative physical therapy (PPT)

Based on experiences in previous studies among physical therapists and patients [14], we will retrospectively evaluate, in the intervention group, the care of the PPT and the compliance of the patients by asking the physical therapists and patients to fill out predefined report forms [14]. The set of questions comprised the survey regarding PPT. In a random sample of 5% we will evaluate the status of the patient of the participating physical therapist what the given PPT care was (content, duration, frequency), and whether this agrees with the case report forms as described above.

In the control group we will also retrospectively evaluate the content, duration and frequency of the PPT with the same report forms.

### Process evaluation

#### Anchor and satisfaction questions

Two anchor questions (“How is your daily functioning changed since your surgery?”/ “How are your pain complaints changed since your surgery?”, a patient satisfaction question on their surgery (“How satisfied are you (in general) about the results of your surgery?”), and a question regarding the given PPT (“How satisfied are you (in general) about the results of your PT treatment?”) will be evaluated at all postoperative time points.

#### (Serious) adverse events & dropout rates

Any (S)AE will be communicated by the local researcher to the central researcher as soon as they become aware of it. The central researchers will report a SAE by the Centrale Commissie Mensgebonden Onderzoek (CCMO) via Toetsingonline within 7 (death or life threatening situations) or 15 (remaining SAE’s) days. All drop-outs and protocol violations will be recorded.

An overview of the assessments can be found in Table 1.

## Sample size and power calculation

Our primary research hypothesis is that the improvement in physical function in our “treat-to-target” intervention will be superior to the improvement in the usual care group at 3 months, measured by the HOOS-PS/KOOS-PS score.

The power calculation is based on the proof of superiority, and calculated for patients undergoing TKA and THA separately. The reported standard deviation (SD) of the KOOS-PS and HOOS-PS 3 months after physiotherapy in TKA and THA patients was 15.6 and 11.8, respectively [35–41]. For the intra cluster correlation coefficient we used an interclass correlation coefficient (ICC) of 0.06, which is generally reported in literature for hospital processes. To detect superiority of the treat-to-target PPT intervention to usual PPT we assessed the required sample size based on the following assumptions, a minimally clinical important difference (MCID) of 10 points, and a SD of 15.6. Since a MCID of the KOOS-PS and HOOS-PS after postoperative physiotherapy in TKA/THA patients is not yet available, a MCID was chosen based on expert opinion, namely 10 points on the KOOS-PS and HOOS-PS [42, 43].

### Primary outcome

We propose a cluster RCT (randomization on hospital level) with an intracluster correlation coefficient of 0.06 and in total 18 participating hospitals. Sample sizes of 90 in group one and 90 in group two, which were obtained by sampling 9 clusters with an average of 10 subjects each in group one (180 patients) and 9 clusters with an average of 10 subjects each in group two (180 patients), achieve 90% power to detect a difference between the group means of at least 10. The coefficient of variation of cluster sizes is 0,500. A two-sided t-test was used with a significance level of 0,05. This test used degrees of freedom based on the number of subjects. To account for a 25% of drop out 240 TKA and 240 THA patients will be needed.

### Secondary outcomes

We also assessed the needed numbers to detect superiority of the treat-to-target PPT intervention to usual PPT for the outcome improvement of pain severity as assessed by the NRS. Based on a MCID of 1 point on the NRS, and a SD of 2 the needed numbers were assessed. Sample sizes of 117 in group one and 117 in group two, which were obtained by sampling 9 clusters with an average of 13 subjects each in group one (234 patients) and 9 clusters with an average of 13 subjects each in group two (234 patients), achieve 91% power to detect a difference between the group means of at least 1. The SD of subjects is 1.7. The intracluster correlation coefficient is 0.06. The coefficient of variation of cluster sizes is 0.500. A two-sided t-test was used with a significance level of 0.05. This test used degrees of freedom based on the number of subjects. To account for a 25% of dropout,624 patients will be needed of which 312 TKA and 312 THA patients.

#### Feasibility of recruitment

Given the planned inclusion period of 12 months and the 18 centers participating and with an expected 100 inclusions a year (~ 2 per week), accounting for patients who are not willing to participate, we expect to have sufficient numbers for our hypothesis. The length of the inclusion period is based on the inclusion rate of the assumed slowest including hospitals (UMCs).

#### Data management

All data are handled confidentially and anonymized in compliance with the Dutch Personal Data Protection Act (“Wet Bescherming Persoonsgegevens”). Questionnaires are collected digitally, and the patient study data are stored in a coded way using secured data management software system Castor EDC [44]. In case an email address is not available, a paper case report form is sent to the patient. When patients fill in their questionnaires on paper, documentation is stored in the investigator site file. Each patient is assigned a random study number that is used for all documentation, study reports and publications. The key to this study number is handled by an independent researcher. All data will be stored during the study period in Castor EDC, after the study is finished the data will be stored for 15 years. Documentation, such as questionnaires filled in on paper and signed informed consent forms, stored in the investigator site file will be stored at each site.

## Statistical analysis

The results will be analyzed separately for TKA and THA. We will analyze patients as treated.

### Primary outcome measure

The difference between both groups in change of KOOS-PS/HOOS-PS between baseline and 3 months follow-up score will be used as primary outcome. Patients will be analyzed as treated.

The primary analyses will be performed by using mixed models (change in KOOS-PS/HOOS-PS during 3 months as dependent and intervention as independent variable). Change between baseline and 3 months follow-up score will be used as primary outcome. Hospital variable will be used to indicate the correlation structure in the model. Adjustments will take place for baseline values of KOOS-PS/HOOS-PS. Of variables of which a priori is known that they are associated with the change in KOOS/HOOS-PS, based on previous studies or based on a strong clinical rationale will be considered as covariates in the primary analysis. These covariates are age, gender, BMI, and surgical approach in THA (anterior, lateral and postero-lateral). The assumptions of constant variance and linear relationships will be assessed. Should any of these assumptions seriously fail then transformation of the dependent or independent variable(s) (where applicable) will be used. The choice of which transformation (e.g. square root, logarithm) will be used will be based on the specific distribution of the residuals.

### Secondary outcomes

By using repeated measures mixed models analyses the course of the secondary outcome(s) over time of both interventions will be compared. The following time points will be used, baseline and follow-up measurements at 6 weeks, 3, 6, 9, 12 months.

Change in secondary outcomes will be used as dependent variable. As secondary outcomes will be used: KOOS-PS/HOOS-PS; OKS/OHS, NRS, AAQ, EuroQol-5D-5L, performance-based test and the anchor and satisfaction questions.

#### Economic evaluation

The economic evaluation will consist of a cost-utility analysis from a societal perspective (costs per QALY), based on patient reports and with an undiscounted one-year time horizon. Average costs and outcomes will be statistically compared using net-benefit analysis, with multiple imputation to account for missing data. Quality-adjusted life years (QALYs) will be estimated using the Dutch tariff for the quarterly EQ-5D-5L measurements. Societal costs will include healthcare costs, patient and family costs, and productivity costs. The timing, contents, frequency and duration of PPT will be measured using the study registrations. Other healthcare use and productivity will be reported by patients using quarterly questionnaires (including general practitioner visits, outpatient visits, hospital days, medication, home and informal care, patient costs, absence from work, and productivity while at (unpaid) work). Healthcare will be valued using reference prices obtained from the Dutch guidelines for economic evaluations in healthcare, including time and travel costs. Sensitivity analysis will be performed on the cost perspective (healthcare instead of societal perspective), the valuation of productivity (human capital instead of friction cost approach) and the utility measure used to calculate QALYs (Visual analogue scale for health (with power transformation) instead of Dutch EQ-5D-5L tariff).

In addition to the CUA, a budget impact analysis (BIA) will be performed. In a cost-calculator spreadsheet model, budget impact will be evaluated from the perspectives of the Dutch ‘Budgettair Kader Zorg’ (BKZ), health insurers, and the different care providers. The analysis will take into account the current mix of treatments, with prices appropriate for the perspective, a 4-year time-horizon and different scenarios for the rate of uptake.

## Discussion

The PATIO trial is, to our knowledge, the first study that analyses the (cost-)effectiveness of a standardized, personalized postoperative physiotherapy protocol compared to usual postoperative care in patients with OA receiving TKA/THA.

Given the large variation in the provision of PPT, which seems to be not or weakly associated with patient characteristics, a more standardized, personalized approach seems warranted. The planned randomized controlled trial, with an economic evaluation will answer the question whether patients who receive a treat-to-target intervention recover with better functional outcomes against lower costs compared to the usual care PPT. From the health care and societal perspective it is extremely relevant that the most optimal PPT treatment strategy is used, as it concerns about 1.66 (THA) and 1.26 (TKA) per 1.000 people per year in western countries [16].

Besides measuring the (cost-) effectiveness of a treat-to-target PPT we also asses a broader range of health aspects (e.g. physical function, pain, comorbidities, perceived health and mental factors, information on insurance and satisfaction level of the results of their surgery and the PT) because several factors can influence the individual recovery after TKA/THA.

Strengths of the study include the nationwide multicenter design, supported by multiple involved professions. PTs are trained to work with the treat-to-target protocol. PTs who are trained on the protocol will unconsciously adapt their usual care treatment. To avoid contamination between the treat-to-target PPT and the usual care PPT a cluster RCT design was chosen. When a PT is trained by the treat-to-target protocol he/she will only treat patients in the intervention group. Since the power and precision of a cluster randomized trial is lower than an individually randomized trial, our study needs more participants to obtain the same statistical power. Another possible limitation of the study is the variation in surgical techniques which may have a different effect in the first months of the revalidation period of the patient.

The results of this study will address an important evidence gap and will have significant impact on the daily practice of the physiotherapist.

## Trial status

The study inclusion has started in August 2018 and is still ongoing.

## Data Availability

The datasets used and/or analyzed during the current study are available from the corresponding author on reasonable request.

## Abbreviations

PTT: Postoperative physical therapy
PT: Physical therapy
PTs: Physical therapist
OA: Osteoarthritis
TKA: Total knee arthroplasty
THA: Total hip arthroplasty
NOV: Dutch Orthopaedic Association
KNGF: the Royal Dutch Society for Physical Therapy
BMI: Body mass index
KOOS: Knee injury and osteoarthritis outcome score
HOOS: Knee disability and osteoarthritis outcome score
OKS: Oxford knee score
OHS: Oxford hip score
NRS: Numeric rating scale (NRS)
NZa: The Dutch Healthcare Authority
BKZ: Dutch Budgettair Kader Zorg
Qaly: Quality adjusted life year
OARSI: Osteoarthritis Research Society International

## References

[1] Westby MD, Brittain A, Backman CL (2014). Expert consensus on best practices for post-acute rehabilitation after total hip and knee arthroplasty: a Canada and United States Delphi study. Arthritis Care Res (Hoboken).

[2] Westby MD, Marshall DA, Jones CA (2018). Development of quality indicators for hip and knee arthroplasty rehabilitation. Osteoarthr Cartil.

[3] Kampshoff dCS, Peter dWFH, MSc MCMvD, Knoop dJ, MSc GAM, Vlieland pdTPMV. KNGF-richtlijn Artrose heup-knie. Conservatieve, pre- en postoperatieve behandeling. 2018.

[4] specialisten Fm. Totale heupprothese (THP) [access date 12-02-2019. Available from: https://richtlijnendatabase.nl/richtlijn/totale_heupprothese_thp/startpagina_-_totale_heup_prothese_thp.html#extra.

[5] Lowe CJM, Barker KL, Dewey M, Sackley CM (2007). Effectiveness of physiotherapy exercise after knee arthroplasty for osteoarthritis: systematic review and meta-analysis of randomised controlled trials. Br Med J.

[6] Minns Lowe CJ, Barker KL, Dewey ME, Sackley CM (2009). Effectiveness of physiotherapy exercise following hip arthroplasty for osteoarthritis: a systematic review of clinical trials. BMC Musculoskelet Disord.

[7] Rahmann AE, Brauer SG, Nitz JC (2009). A specific inpatient aquatic physiotherapy program improves strength after total hip or knee replacement surgery: a randomized controlled trial. Arch Phys Med Rehabil.

[8] Liebs TR, Herzberg W, Ruther W, Haasters J, Russlies M, Hassenpflug J (2010). Ergometer cycling after hip or knee replacement surgery: a randomized controlled trial. J Bone Joint Surg Am.

[9] Roos EM (2003). Effectiveness and practice variation of rehabilitation after joint replacement. Curr Opin Rheumatol.

[10] Westby MD (2012). Rehabilitation and total joint arthroplasty. Clin Geriatr Med.

[11] Peter WF, Nelissen RG, Vlieland TP (2014). Guideline recommendations for post-acute postoperative physiotherapy in total hip and knee arthroplasty: are they used in daily clinical practice?. Musculoskeletal Care..

[12] Artz N, Dixon S, Wylde V, Beswick A, Blom A, Gooberman-Hill R (2013). Physiotherapy provision following discharge after total hip and total knee replacement: a survey of current practice at high-volume NHS hospitals in England and wales. Musculoskeletal Care..

[13] Chen H (2012). Ea. association between rehabilitation timing and major complications of total knee arthroplatsy. J Rehabil Med.

[14] Peter WF, Tilbury C, Verdegaal SHM, Onstenk R, Vehmeijer SB, Vermeulen EM (2016). The provision of preoperative and postoperative physical therapy in elderly people with hip and knee osteoarthritis undergoing primary joint replacement surgery. Curr Orthop Pract.

[15] Wijnen A, Bouma SE, Seeber GH, van der Woude LHV, Bulstra SK, Lazovic D (2018). The therapeutic validity and effectiveness of physiotherapeutic exercise following total hip arthroplasty for osteoarthritis: a systematic review. PLoS One.

[16] Health at a Glance: OECD Indicators, OECD Publishing; 2017 [Available from: https://www.oecd-ilibrary.org/social-issues-migration-health/health-at-a-glance-2017_health_glance-2017-en;jsessionid=sHVPNloegsvfCp0tRJdc6BR7.ip-10-240-5-110. Accessed 10 Nov 2017.

[17] (LROI) DAR. Online LROI annual report 2018. LROI; 2018. http://www.lroi-rapportage.nl/media/pdf/PDF%20Online_LROI_annual_report_2018.pdf:. Accessed Aug 2018.

[18] Lewiecki EM (2003). Nonresponders to osteoporosis therapy. J Clin Densitom.

[19] van de Sant AJW, de Vries NM, Hoogeboom TJ (2019). Nijhuis-van der Sanden MWG. Implementation of a personalized, cost-effective physical therapy approach (Coach2Move) for older adults: barriers and facilitators. J Geriatr Phys Ther.

[20] Heij W, Teerenstra S, Sweerts L, Staal B, der Sanden MWG N-v, Hoogeboom TJ. Implementation of a cost-effective physical therapy approach (Coach2Move) to improve physical activity in community-dwelling older adults with mobility problems: Protocol for a Cluster-Randomized, Stepped Wedge Trial. Phys Ther. 2020;100(4):653–61. 10.1093/ptj/pzz183 PMC7297439 31846501

[21] Featherall J, Brigati DP, Arney AN, Faour M, Bokar DV, Murray TG (2019). Effects of a Total knee Arthroplasty care pathway on cost, quality, and patient experience: toward measuring the triple aim. J Arthroplast.

[22] Hoeffel DP, Daly PJ, Kelly BJ, Giveans MR (2019). Outcomes of the first 1,000 Total hip and Total knee Arthroplasties at a same-day surgery center using a rapid-recovery protocol. J Am Acad Orthop Surg Glob Res Rev.

[23] Kimball CC, Nichols CI, Vose JG. Outpatient versus rapid recovery inpatient knee Arthroplasty: comparison of matched cohorts. Orthopedics. 2020;43(1):36–41. 10.3928/01477447-20191122-01 31770445

[24] de Groot IB, Reijman M, Terwee CB, Bierma-Zeinstra SM, Favejee M, Roos EM (2007). Validation of the Dutch version of the hip disability and osteoarthritis outcome score. Osteoarthr Cartil.

[25] Roos EM, Lohmander LS (2003). The knee injury and osteoarthritis outcome score (KOOS): from joint injury to osteoarthritis. Health Qual Life Outcomes.

[26] Roos EM, Roos HP, Lohmander LS, Ekdahl C, Beynnon BD (1998). Knee injury and osteoarthritis outcome score (KOOS)--development of a self-administered outcome measure. J Orthop Sports Phys Ther.

[27] de Groot IB, Favejee MM, Reijman M, Verhaar JA, Terwee CB (2008). The Dutch version of the knee injury and osteoarthritis outcome score: a validation study. Health Qual Life Outcomes.

[28] Hakkaart-van Roijen L, van Straten A, Donker M, Tiemens B. Handleiding Trimbos/iMTA questionnaire for Costs associated with Psychiatric illness (TiC-P). Insituut voor Medische Technology Assessment, Erasmus University Rotterdam, Trimbos. 2002.

[29] Dobson F, Hinman RS, Roos EM, Abbott JH, Stratford P, Davis AM (2013). OARSI recommended performance-based tests to assess physical function in people diagnosed with hip or knee osteoarthritis. Osteoarthr Cartil.

[30] McCaffery M (2001). Using the 0-to-10 pain rating scale. Am J Nurs.

[31] Gosens T, Hoefnagels NH, de Vet RC, Dhert WJ, van Langelaan EJ, Bulstra SK (2005). The “Oxford Heup score”: the translation and validation of a questionnaire into Dutch to evaluate the results of total hip arthroplasty. Acta Orthop.

[32] Haverkamp D, Breugem SJ, Sierevelt IN, Blankevoort L, van Dijk CN (2005). Translation and validation of the Dutch version of the Oxford 12-item knee questionnaire for knee arthroplasty. Acta Orthop.

[33] Peter WF, de Vet HCW, Terwee CB (2018). Reliability of the animated activity questionnaire for assessing activity limitations of patients with hip and knee osteoarthritis. Musculoskeletal Care.

[34] Rabin R, de Charro F (2001). EQ-5D: a measure of health status from the EuroQol group. Ann Med.

[35] Beaupre LA, Masson EC, Luckhurst BJ, Arafah O, O'Connor GJ (2014). A randomized pilot study of a comprehensive postoperative exercise program compared with usual care following primary total hip arthroplasty in subjects less than 65 years of age: feasibility, selection of outcome measures and timing of assessment. BMC Musculoskelet Disord.

[36] Skoffer B, Dalgas U, Mechlenburg I (2015). Progressive resistance training before and after total hip and knee arthroplasty: a systematic review. Clin Rehabil.

[37] Papalia R, Vasta S, Tecame A, D'Adamio S, Maffulli N, Denaro V (2013). Home-based vs supervised rehabilitation programs following knee surgery: a systematic review. Br Med Bull.

[38] Coulter CL, Scarvell JM, Neeman TM, Smith PN (2013). Physiotherapist-directed rehabilitation exercises in the outpatient or home setting improve strength, gait speed and cadence after elective total hip replacement: a systematic review. J Physiother.

[39] Mikkelsen LR, Mechlenburg I, Soballe K, Jorgensen LB, Mikkelsen S, Bandholm T (2014). Effect of early supervised progressive resistance training compared to unsupervised home-based exercise after fast-track total hip replacement applied to patients with preoperative functional limitations. A single-blinded randomised controlled trial. Osteoarthr Cartil.

[40] Moffet H, Tousignant M, Nadeau S, Merette C, Boissy P, Corriveau H (2015). In-home Telerehabilitation compared with face-to-face rehabilitation after Total knee Arthroplasty: a noninferiority randomized controlled trial. J Bone Joint Surg Am.

[41] Buker N, Akkaya S, Akkaya N, Gokalp O, Kavlak E, Ok N (2014). Comparison of effects of supervised physiotherapy and a standardized home program on functional status in patients with total knee arthroplasty: a prospective study. J Phys Ther Sci.

[42] Skou ST, Roos EM, Laursen MB, Rathleff MS, Arendt-Nielsen L, Simonsen O (2015). A randomized, controlled trial of Total knee replacement. N Engl J Med.

[43] Celik D, Coban O, Kilicoglu O (2019). Minimal clinically important difference of commonly used hip-, knee-, foot-, and ankle-specific questionnaires: a systematic review. J Clin Epidemiol.

[44] Castor EDC. (2020). Castor electronic data capture. [online] Available at: https://castoredc.com.

